# Supplementary material for: Encrypt with Your Mind: Reliable and Revocable Brain Biometrics via Multidimensional Gaussian Fitted Bit Allocation
Source: Bioengineering (Basel). 2023 Aug 1;10(8):912. doi: 10.3390/bioengineering10080912 (PMC10451328; doi:10.3390/bioengineering10080912)
Supplement: Supplementary file 1 [file bioengineering-10-00912-s001.zip › bioengineering-2474498-supplementary.pdf]

Supplementary Material of Encrypt with Your Mind: Reliable and Revocable Brain Biometrics via Multidimensional Gaussian Fitted Bit Allocation

Ming Li et al.

The exact 96 feature distribution of 10 rats are as follows:

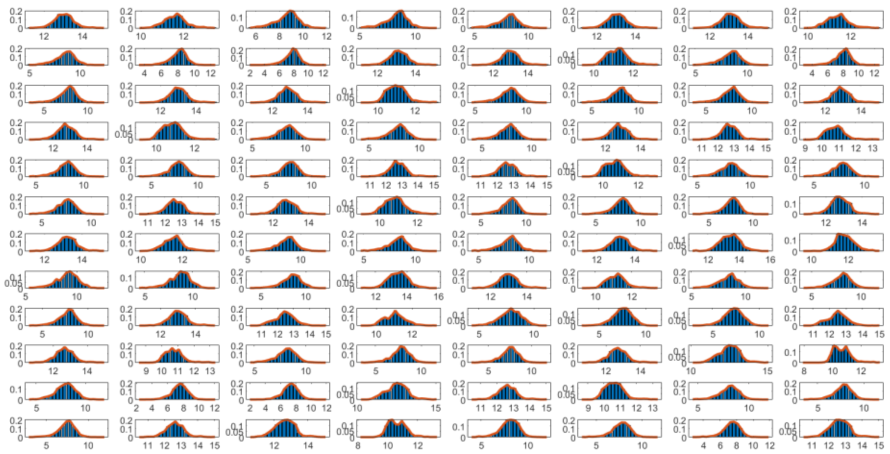

Figure S1. The exact 96 feature distribution of Rat-1

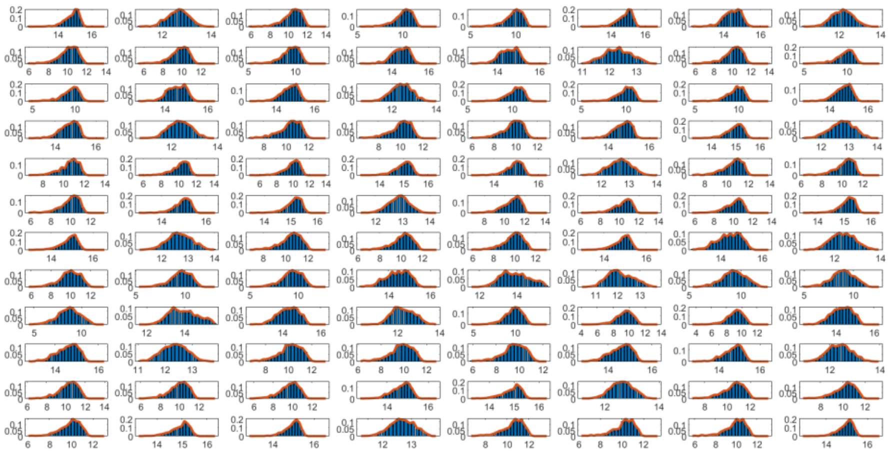

Figure S2. The exact 96 feature distribution of Rat-2

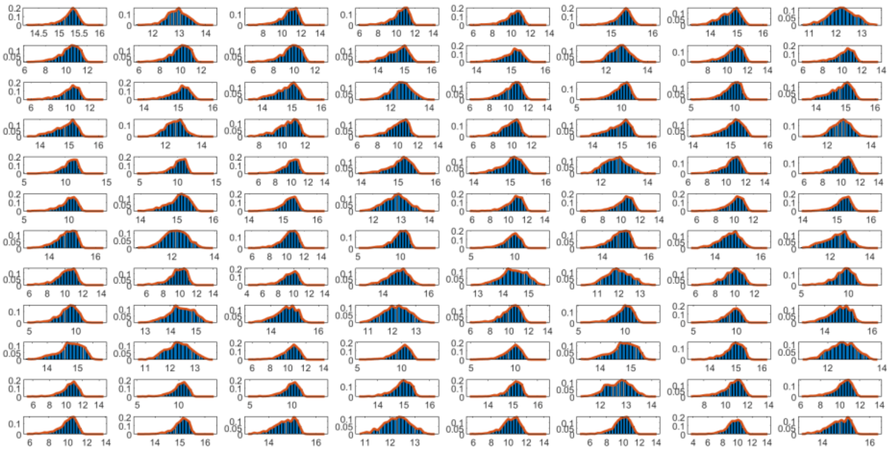

Figure S3. The exact 96 feature distribution of Rat-3

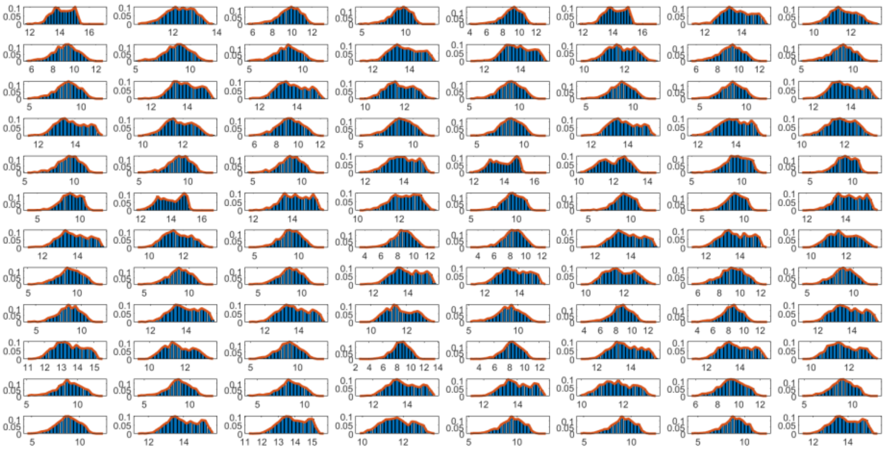

Figure S4. The exact 96 feature distribution of Rat-4

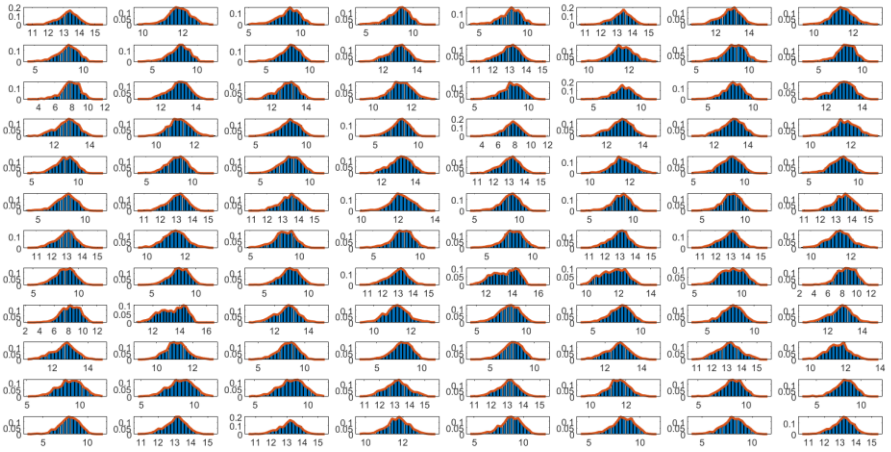

Figure S5. The exact 96 feature distribution of Rat-5

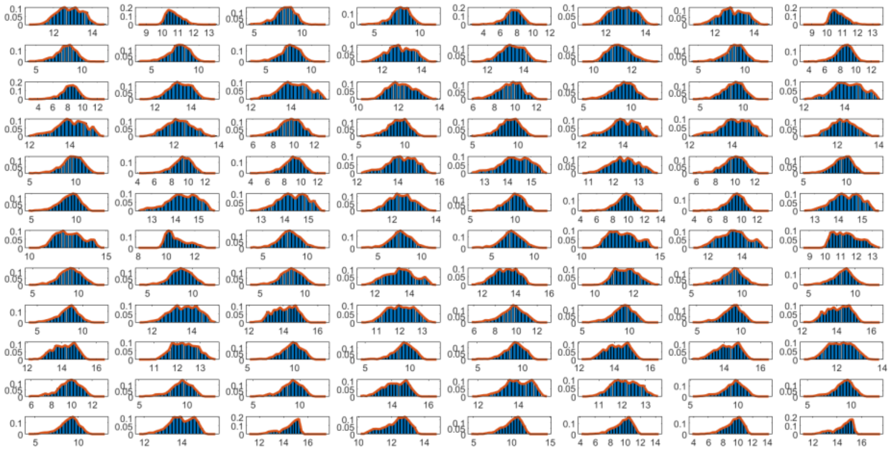

Figure S6. The exact 96 feature distribution of Rat-6

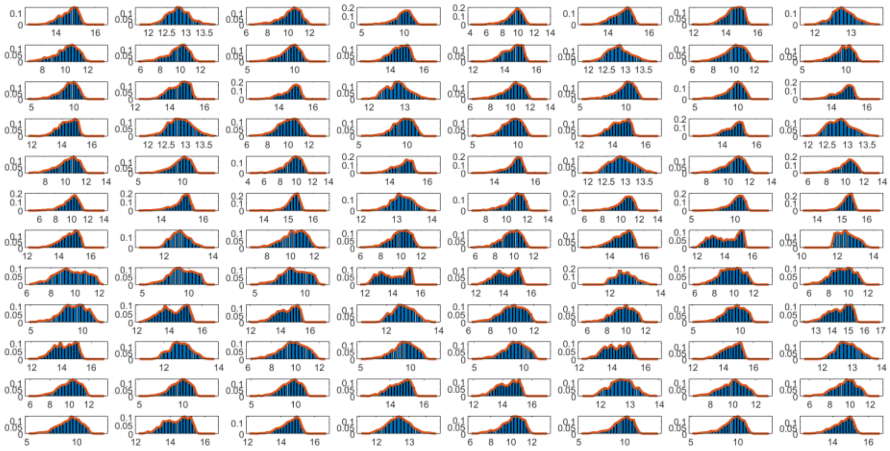

Figure S7. The exact 96 feature distribution of Rat-7

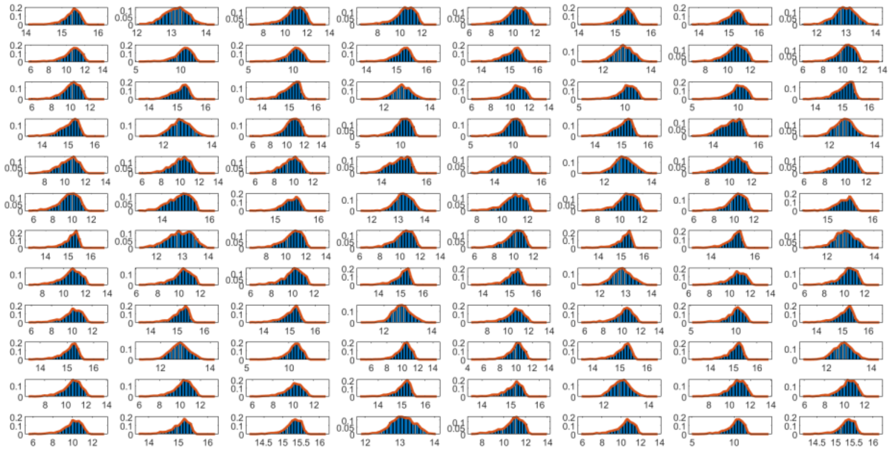

Figure S8. The exact 96 feature distribution of Rat-8

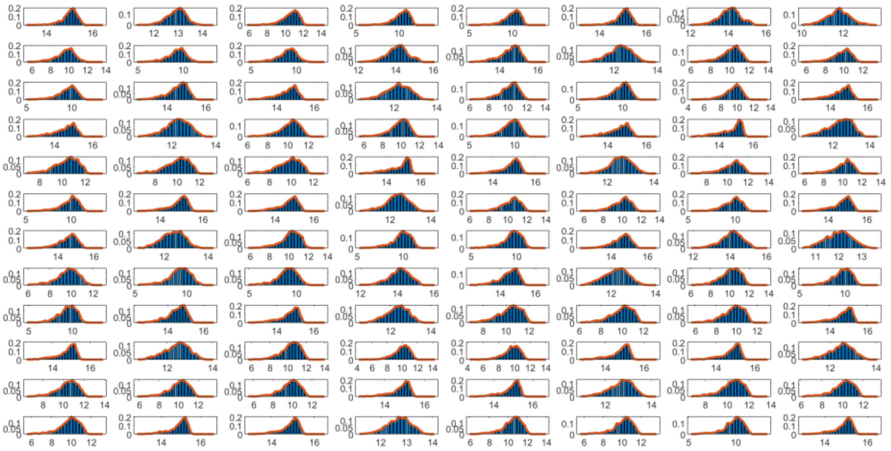

Figure S9. The exact 96 feature distribution of Rat-9

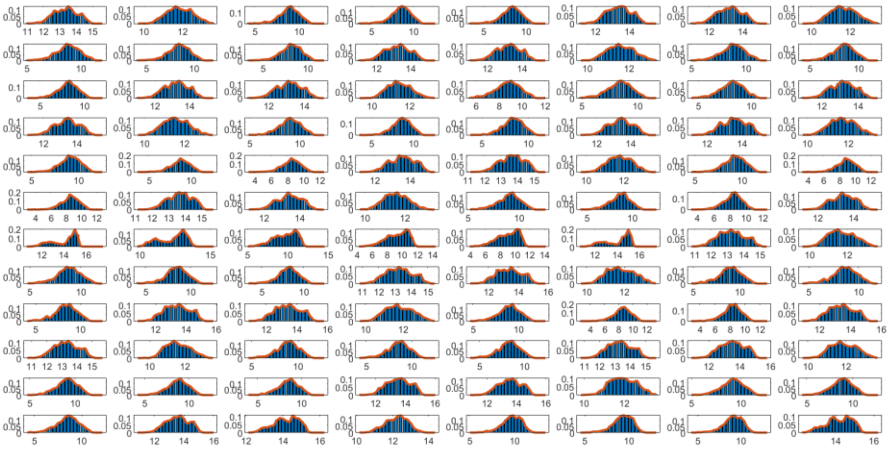

Figure S10. The exact 96 feature distribution of Rat-10

Table S1. The identification results with different classifiers using 96-dim features:

Classifier	Identification Accuracy (10 rats)
Linear Discriminant Analysis	0.679
Decision Tree	0.362
k-nearest neighbor	0.341
Support Vector Machine	0.722
